# Regorafenib reverses HGF‐induced sorafenib resistance by inhibiting epithelial‐mesenchymal transition in hepatocellular carcinoma

**DOI:** 10.1002/2211-5463.12578

**Published:** 2019-01-18

**Authors:** Weibo Chen, Junsheng Yang, Yue Zhang, Huihua Cai, Xuemin Chen, Donglin Sun

**Affiliations:** ^1^ Department of Hepatopancreatobiliary Surgery the Third Affiliated Hospital of Soochow University Changzhou China

**Keywords:** EMT, HGF, regorafenib, Snail, sorafenib resistance

## Abstract

Sorafenib resistance is one of the major obstacles towards achieving a better outcome in patients with advanced hepatocellular carcinoma (HCC), in which aberrant activation of the hepatocyte growth factor (HGF)/mesenchymal‐epithelial transition pathway is frequently observed. Here, we report that HCC cells develop sorafenib resistance following HGF stimulation. Furthermore, HGF activates the downstream extracellular signal‐related kinase (ERK) and signal transducer and activator of transcription 3 (STAT3) pathway and induces epithelial–mesenchymal transition (EMT) by up‐regulating Snail in HCC cells. Inhibition of ERK and STAT3 abolished the rescue effect of HGF by down‐regulating Snail and EMT. Moreover, phosphoinositide 3‐kinase/Akt was also activated in HGF‐treated HCC cells, although it had no effect on Snail expression. Notably, we also found that regorafenib reversed HGF‐induced sorafenib resistance by inhibiting ERK and STAT3, and subsequently down‐regulating Snail and EMT. Taken together, our results indicate that HGF induces sorafenib resistance by activating phosporylated (P)‐ERK/Snail/EMT and P‐STAT3/Snail/EMT pathways. Inhibition of P‐ERK and P‐STAT3 by regorafenib can block HGF‐induced EMT, thereby reversing HGF‐induced sorafenib resistance.

**Abbreviations**
EMT: epithelial‐mesenchymal transition
HCC: hepatocellular carcinoma
HGF: hepatocyte growth factor
MEK: mitogen‐activated protein kinase kinase
MET: mesenchymal‐epithelial transition
PARP: poly ADP‐ribose polymerase
PI3K: phosphoinositide 3‐kinase
RAF: rapidly accelerated fibrosarcoma
SHP‐1: X (RFX)/SH2 domain‐containing phosphatase 1
siRNA: small interfering RNA
STAT3: signal transducer and activator of transcription 3

Hepatocellular carcinoma (HCC) is the fifth most commonly‐diagnosed cancer and is the second leading cause of cancer‐related death around the world. More than 780 000 new cases and 740 000 deaths were reported to occur worldwide annually 1. HCC is treated by the surgery oriented comprehensive therapy; however, over 80% of HCC cases are diagnosed at advanced stages and lost surgery opportunities 2. For late‐stage HCC patients, chemotherapy shows low reactivity with treatment‐related toxicity and provides no survival benefits 3. Thus, effective palliative therapies are in urgent demand 4.

Sorafenib is the first systemic therapy approved by the Food and Drug Administration to demonstrate a survival benefit with adequate safety profile for unresectable HCC 5. It improves overall survival by 2.8 months on average and postpones time‐to‐radiological disease progression 6. However, the total response rate on sorafenib is only approximately 30% and HCC often progresses within 6 months, suggesting that innate and acquired sorafenib resistance exists in HCC cells 5, 6. Many studies have reported findings on the mechanisms underlying sorafenib resistance, such as epithelial–mesenchymal transition (EMT) and the tumor microenvironment, which have tight relationships and coordinate the resistance 7. When cancer cells undergo EMT, epithelial cells lose cell‐to‐cell interaction and acquire mesenchymal properties. The role of EMT in cancer metastasis and recurrence remains controversial, although it is confirmed that cancer cells develop resistance after EMT 8, 9. It has been demonstrated that resistance to targeted therapy can be conferred by tumor microenvironment in which soluble factors, such as cytokines and growth factors secreted by stromal cells, play decisive roles 10, 11. Our preliminary research showed that hepatocyte growth factor (HGF) secreted by hepatic stellate cells could attenuate sorafenib‐induced cell death in HCC cells by activating the HGF/mesenchymal‐epithelial transition (MET) axis 12. However, it remains to be determined whether HGF triggers sorafenib resistance by inducing EMT in HCC cells.

Regorafenib is the only systemic therapy shown to provide survival advantages in HCC patients progressing on sorafenib treatment. In a certain group of HCC patients who tolerated sorafenib, progressed on sorafenib, and had Child–Pugh A liver function, regorafenib is reported to improve the median survival by 2.8 months compared to placebo 13. Regorafenib has been approved by the Food and Drug Administration as second‐line therapy in HCC 14. In HCC, regorafenib induces significant tumor inhibition and induces extrinsic and intrinsic apoptosis via the inhibition of signal transducer and activator of transcription 3 (STAT3) and the extracellular signal‐related kinase (ERK)/nuclear factor‐kappa B (NF&kgr;B) pathways 15, 16, 17. However, the role of regorafenib in EMT and sorafenib resistance of HCC has not yet been reported.

In the present study, we aimed to determine whether HGF triggered sorafenib resistance in HCC cells by inducing EMT and whether regorafenib had an inhibitory role on HGF‐induced sorafenib resistance.

## Materials and methods

### Cell culture and reagents

Human HCC cell SMMC‐7721 was purchased from Cell Bank of Xiangya Central Laboratory, Central South University. HepG2 was obtained from the Cell Bank of Shanghai Institute of Biological Sciences, Chinese Academy of Sciences (Shanghai, China). They were maintained in Dulbecco's modified Eagle's medium (DMEM) (Gibco, Gaithersburg, MD, USA) supplemented with 10% fetal bovine serum (FBS) (Gibco) at 37 °C in a humidified atmosphere with 5% CO_2_. Sorafenib tosylate, regorafenib (BAY 73‐4506), U0126, MK2206 2 HCl and S3I‐201 were purchased from Selleck (Selleck Chemicals, Shanghai, China). Recombinant human HGF protein was purchased from R&D Systems (Minneapolis, MN, USA). Primary antibodies against PARP (9532), Snail (3879), E‐cadherin (3195), vimentin (5741), α‐tubulin (2144), MET (8198), phosphorylated (P)‐MET (3077), ERK (4695), P‐ERK (4370), Akt (2920), P‐Akt (4060), STAT3 (12640) and P‐STAT3 (9145) were purchased from Cell Signaling Technology (Beverly, MA, USA). The primary antibody against GAPDH (AB22131) was obtained from Bioworld Technology, Inc. (St Louis Park, MN, USA).

### CCK‐8 assay

Cell viability was monitored using CCK‐8 assay. Generally, HCC cells seeded in 96‐well plates were incubated with CCK‐8 (Dojindo Laboratories, Kumamoto, Japan) for 2 h. The absorbance values at 490 nm were measured using a microplate reader (Thermo Fisher Scientific, Waltham, MA, USA). All experiments were performed in triplicate.

### Flow cytometry

Cell apoptosis was assessed by flow cytometry as described previously 18. In brief, after being harvested and collected by centrifugation, HCC cells were washed with phosphate‐buffered saline and resuspended in 500 μL of binding buffer. Then, 5 μL of annexin V‐fluorescein isothiocyanate and 5 μL of propidium iodide (Beyotime, Nantong, China) were added. The cells were incubated in the dark for 10 min and then subjected to flow cytometric analysis.

### Western blotting

Total cell lysates were prepared by NP‐40 solution containing protease inhibitors. Cell protein extracts were boiled in loading buffer and subjected to SDS/PAGE. Then, the protein was transferred onto polyvinylidene difluoride membranes and incubated with indicated primary antibodies overnight at 4 °C. Following washing with Tris‐buffered saline and Tween 20, the membranes were incubated with horseradish peroxidase‐conjugated second antibody for 2 h at room temperature. The signals were developed using an enhanced chemiluminescence system (Merck Millipore, Schaffhausen, Switzerland) and captured on X‐OMAT BT films (Carestream, Shanghai, China). The density of western blotting bands was analyzed using image j, version 1.8.0 (NIH, Bethesda, MD, USA).

### Quantitative RT‐PCR

Total RNA was extracted using TRIzol® reagent (Takara Bio Inc., Otsu, Japan) and reverse‐transcribed to cDNA using PrimeScript™ RT Master Mix (Takara Bio Inc.). The relative mRNA expression levels were determined by a quantitative RT‐PCR with SYBR® Premix Ex Taq™ PCR kit (Takara Bio Inc.) on an ABI PRISM® 7300 Sequence Detection System (Applied Biosystems, Foster City, CA, USA). The relative mRNA levels were calculated by the 2^−ΔΔCq^ method with GAPDH as the internal control. The sequences of the primers used were: *snail* (forward) 5′‐TCGGAAGCCTAACTACAGCGA‐3′, *snail* (reverse) 5′‐AGATGAGCATTGGCAGCGAG‐3′; *slug* (forward) 5′‐CGAACTGGACACACATACAGTG‐3′, *slug* (reverse) 5′‐CTGAGGATCTCTGGTTGTGGT‐3′; *twist1* (forward) 5′‐GTCCGCAGTCTTACGAGGAG‐3′, *twist1* (reverse) 5′‐GCTTGAGGGTCTGAATCTTGCT‐3′; *zeb1* (forward) 5′‐GATGATGAATGCGAGTCAGATGC‐3′, *zeb1* (reverse) 5′‐ACAGCAGTGTCTTGTTGTTGT‐3′; *zeb2* (forward) 5′‐CAAGAGGCGCAAACAAGCC‐3′, *zeb2* (reverse) 5′‐GGTTGGCAATACCGTCATCC‐3′; GAPDH (forward) 5′‐CTCACCGGATGCACCAATGTT‐3′, GAPDH (reverse) 5′‐CGCGTTGCTCACAATGTTCAT‐3′.

### Wound healing assay

The wound healing assay was performed using Wound Healing Culture‐inserts (Ibidi, Munich, Germany) to measure the migration capacity of tumor cells. In brief, 35 000 cells were seeded in each well of the culture‐insert and incubated for 24 h. Thereafter, the culture‐insert was removed to generate cell‐free area with the width of approximately 0.5 mm. The cells were cultured in FBS‐free DMEM for indicated time and the migration was captured under an BX51 microscope (Olympus, Tokyo, Japan). The wound closure rate was calculated.

### Transwell assay

The transwell assay was performed using Transwell inserts (Merck Millipore). In brief, the upper chamber membrane was coated with Matrigel (354230) (Becton‐Dickinson Biosciences, Franklin Lakes, NJ, USA) for 30 min at 37 °C and then was added with DMEM to hydrate the membrane for 30 min. Next, 50 000 HCC cells resuspended in DMEM were seeded to the upper chamber. The lower chamber was added with DMEM supplemented with 10% FBS. After being cultivated for indicated time, the upper chamber membrane was fixed in ice‐cold methanol. Cells on the opposite side of the membrane were stained with crystal violet and photographed and counted under an BX51 microscope (Olympus).

### Small interfering RNA transfection

The human *snail*‐siRNA (sc‐38398) and control‐siRNA (sc‐37007) were purchased from Santa Cruz Biotechnology, Inc. (Santa Cruz, CA, USA). The transfection was conducted using Lipofectamine® RNAiMAX (Thermo Fisher Scientific) in accordance with the manufacturer's instructions.

### Statistical analysis

All of the data are presented as the mean ± SD. Data were analyzed using Student's *t*‐test and two‐way ANOVA with Bonferroni correction. The calculations were performed using spss, version 16.0 (SPSS Inc., Chicago, IL, USA). The statistical survival analysis was performed using spss. *P* < 0.05 was considered statistically significant.

## Results

### HGF induces sorafenib resistance in HCC cells

**Figure 1:**
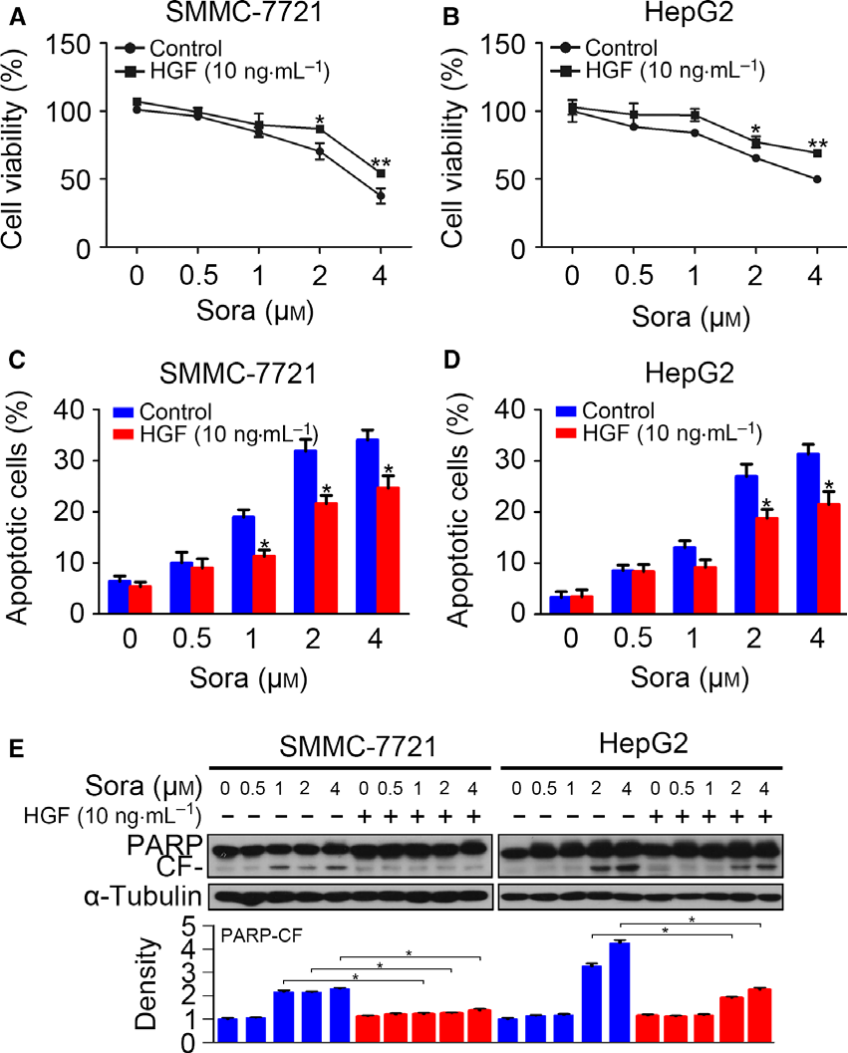
Hepatocyte growth factor induces sorafenib resistance in HCC cells. (A and B) Serum‐starved SMMC‐7721 and HepG2 cells were incubated with sorafenib for 48 h after pre‐treatment with HGF (10 ng·mL^−1^) for 24 h. Cell viability was detected by CCK‐8 assay. (C and D) Treatment was as described above, and the apoptotic cells were determined by flow cytometry. (E) Treatment was as described above, and cell apoptosis was detected by western blotting of PARP. The density of each band was normalized to α‐tubulin and is displayed below. CF, cleaved form. (*P < 0.05, **P < 0.01). Data are expressed as the mean ± SD from three individual experiments. Differences between groups were determined using Student's *t*‐test and two‐way ANOVA with Bonferroni correction.

To investigate whether HGF induced sorafenib resistance in HCC cells, SMMC‐7721 and HepG2 cells were pre‐incubated with HGF of 10 ng·mL^−1^ for 24 h, and cell viability was determined via CCK‐8 after administration of sorafenib for 48 h. We found that sorafenib inhibited cell viability in both cell lines, whereas pre‐treatment of HGF attenuated the inhibition (Fig. 1A,B). Then, using a flow cytometry assay, we analyzed the sorafenib‐induced apoptosis with or without HGF stimulation. We found cell apoptosis in sorafenib‐treated HCC cells, whereas HGF treatment decreased the amount of apoptotic cells (Fig. 1C,D). To further confirm that HGF pre‐incubation inhibited cell apoptosis, we detected the protein level of poly ADP‐ribose polymerase (PARP) and cleaved PARP in HCC cells following the treatment described above. We observed a decrease in the cleaved form of PARP in both cell lines (Fig. 1E). These results confirm that pre‐treatment of HGF induced sorafenib resistance in HCC cells.

### HGF induces EMT by up‐regulating Snail in HCC cells

**Figure 2:**
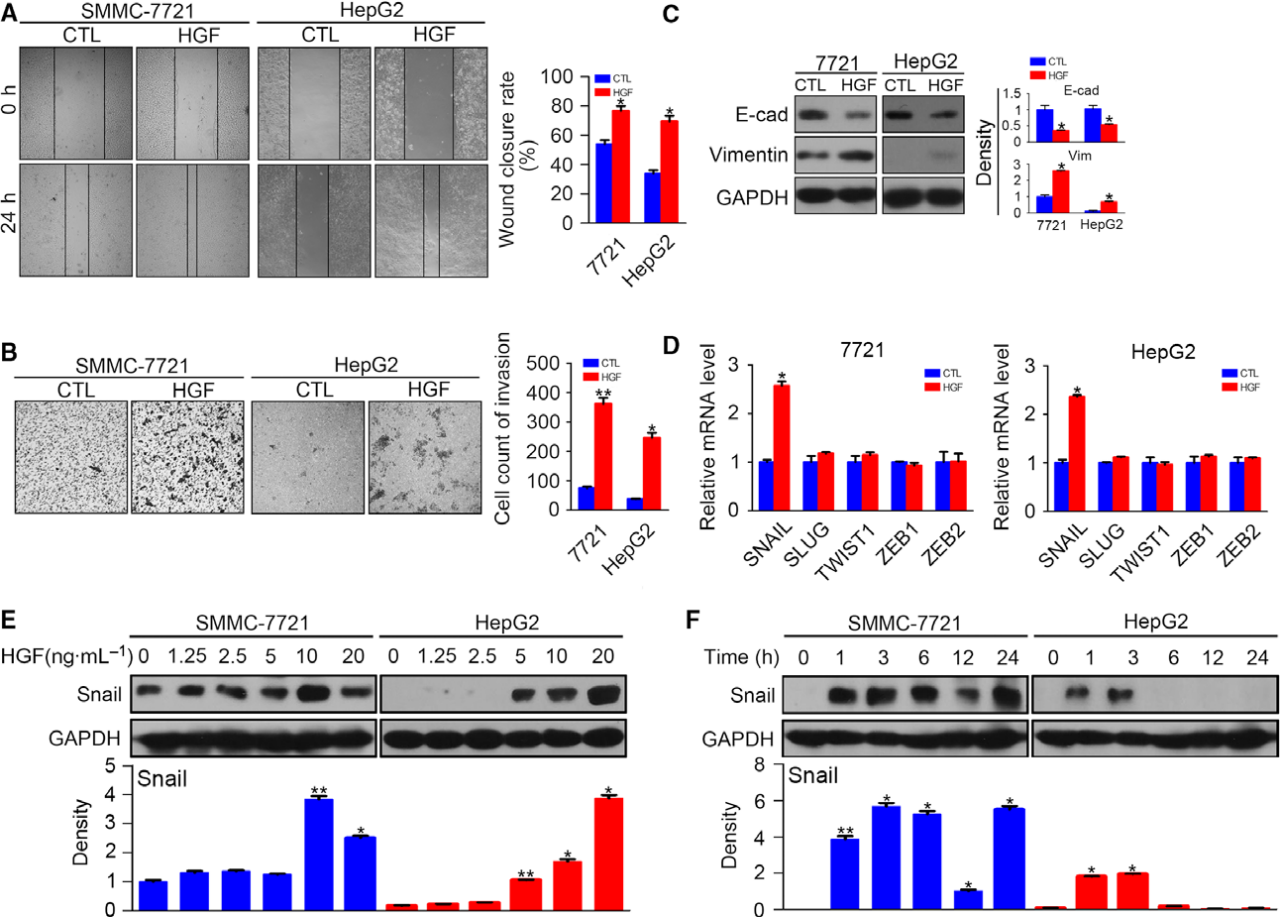
Hepatocyte growth factor induces EMT by up‐regulating Snail in HCC cells. (A) Serum‐starved SMMC‐7721 and HepG2 were stimulated with or without HGF (10 ng·mL^−1^) for 24 h, and then cell migration was determined by a wound healing assay. (B) Serum‐starved SMMC‐7721 and HepG2 were stimulated with or without HGF (10 ng·mL^−1^) for 24 h, and then cell invasion was determined by a transwell assay. (C) Serum‐starved SMMC‐7721 and HepG2 were stimulated with or without HGF (10 ng·mL^−1^) for 48 h, and the protein levels of E‐cadherin and vimentin were detected by western blotting. The density of each band was normalized to GAPDH. (D) Quantitative RT‐PCR results of *snail*,* slug*,* twist1*,* zeb1* and *zeb2* after incubation with HGF for 3 h. (E) Serum‐starved SMMC‐7721 and HepG2 were stimulated with HGF at different concentrations for 3 h, and protein levels of Snail were detected by western blotting. The density of each band was normalized to GAPDH. (F) Serum‐starved SMMC‐7721 and HepG2 were stimulated with HGF (10 ng·mL^−1^) for different times, and protein levels of Snail were detected by western blotting. The density of each band was normalized to GAPDH. (*P < 0.05, **P < 0.01, compared to control). Data are expressed as the mean ± SD from three individual experiments. Differences between groups were determined using Student's *t*‐test and two‐way ANOVA with Bonferroni correction.

A previous study revealed that HGF induced EMT in HCC cells 19 and EMT was found to play a critical role in cancer drug resistance 8, 9; thus, we focused on EMT in the present study. We examined the migration and invasion of HCC cells after HGF stimulation. We found that HGF enhanced cell migration and invasion in both cell lines (Fig. 2A,B). HGF administration down‐regulated the epithelial marker E‐cadherin and up‐regulated the mesenchymal marker vimentin at the protein level (Fig. 2C). Next, we evaluated the mRNA changes of EMT transcription factors and found that only *snail* was up‐regulated in both HCC cell lines after incubation with HGF for 3 h (Fig. 2D). This result was consistent a study reported by Nagai *et al*. 19 reporting that HGF up‐regulated Snail and induced EMT in HCC cells. We also found that HGF increased Snail protein levels dose‐dependently after 3 h of incubation (Fig. 2E). The time‐course analysis shows that HGF started to increased Snail expression at 1 h after stimulation. The protein level of Snail recovered to the baseline at 6 h in HepG2 cells; however, the Snail level did not decrease in SMMC‐7721 cells, even at 24 h (Fig. 2F).

### Silencing of *snail* reverses HGF‐induced sorafenib resistance

**Figure 3:**
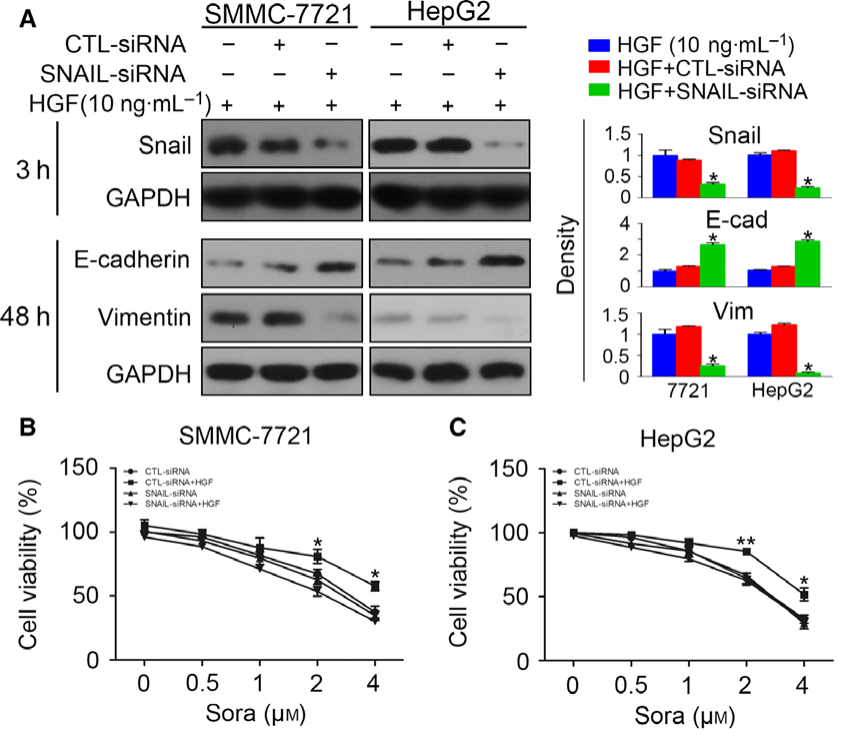
Silencing of *snail* reverses HGF‐induced sorafenib resistance. (A) SMMC‐7721 and HepG2 cells transfected with CTL‐siRNA or *snail*‐siRNA were incubated with HGF (10 ng·mL^−1^) and protein levels of Snail (3 h after incubation), E‐cadherin and vimentin (48 h after incubation) were detected. The density of each band was normalized to GAPDH (*P < 0.05, compared to HGF). (B and C) SMMC‐7721 and HepG2 cells transfected with CTL‐siRNA or *snail*‐siRNA were incubated with sorafenib with or without HGF pre‐treatment (10 ng·mL^−1^) and cell viability was detected by the CCK‐8 assay (*P < 0.05, **P < 0.01, CTL‐siRNA+HGF vs. *snail*‐siRNA+HGF). Data are expressed as the mean ± SD from three individual experiments. Differences between groups were determined using Student's *t*‐test and two‐way ANOVA with Bonferroni correction.

To determine whether the induced EMT was responsible for sorafenib resistance, we adopted *snail* siRNA to block the *snail* increase in HCC cells. The interfering efficiency was first confirmed by western blotting, which showed that transfection of *snail* siRNA reversed the increase of Snail after HGF stimulation for 3 h at the protein level. Then, we detected the protein level of E‐cadherin and vimentin in HCC cells after siRNA transfection. The silencing of *snail* inhibited the down‐regulation of E‐cadherin and the up‐regulation of vimentin (Fig. 3A), which confirmed that down‐regulation of *snail* reversed HGF‐induced EMT in HCC cells. To clarify whether the inhibition of EMT could reverse sorafenib resistance, HCC cells with *snail* knockdown were pre‐treated with HGF and incubated with sorafenib for 48 h. The CCK‐8 assay demonstrated that transfection of *snail* siRNA inhibited the protective role of EMT on cell viability (Fig. 3B,C), indicating that inhibition of EMT reversed HGF‐induced sorafenib resistance.

### Inhibition of HGF/MET signaling reverses EMT and sorafenib resistance

**Figure 4:**
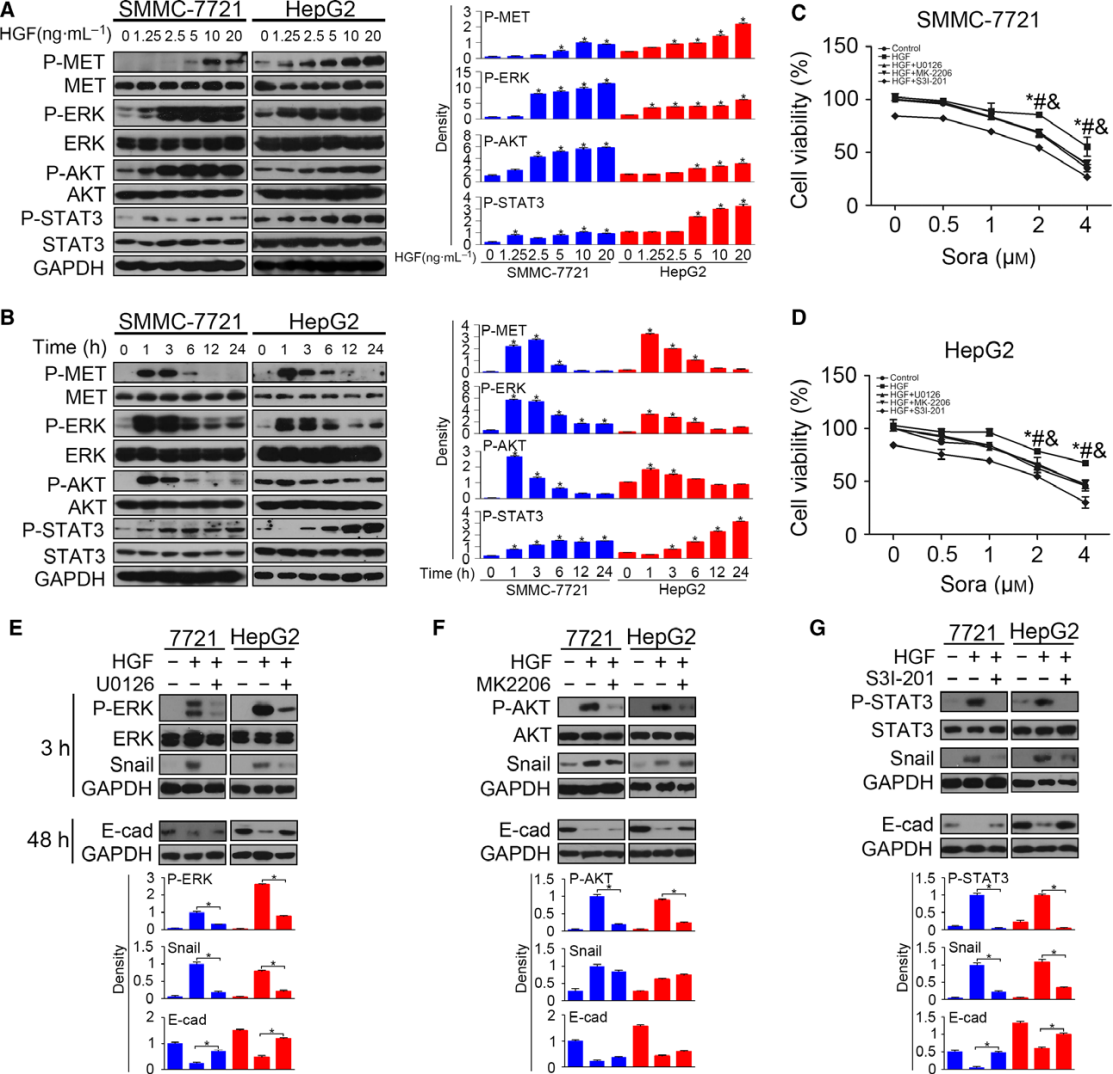
Inhibition of HGF/MET signaling reverses EMT and sorafenib resistance. (A) Serum‐starved SMMC‐7721 and HepG2 were stimulated with HGF for 1 h, and the activated P‐MET, P‐ERK, P‐AKT and P‐STAT3 were examined by western blotting. The density of each band was normalized to GAPDH (*P < 0.05, compared to control). (B) Serum‐starved SMMC‐7721 and HepG2 were stimulated with HGF (10 ng·mL^−1^) for different times and then the activated P‐MET, P‐ERK, P‐AKT and P‐STAT3 were examined by western blotting. The density of each band was normalized to GAPDH (*P < 0.05, compared to control). (C and D) Serum‐starved SMMC‐7721 and HepG2 pre‐incubated with U0126 (2 μm)/MK2206 (2 μm)/S3I‐201 (160 μm) for 6 h were treated with HGF (10 ng·mL^−1^) for 24 h and, thereafter, cells were incubated with sorafenib for 48 h. Cell viability was accessed by the CCK‐8 assay (*P < 0.05, HGF + U0126 vs. HGF; #*P* < 0.05, HGF + MK2206 vs. HGF; &*P* < 0.05, HGF + S3I‐201 vs. HGF). (E) Serum‐starved SMMC‐7721 and HepG2 cells pre‐incubated with U0126 were treated with HGF (10 ng·mL^−1^) and P‐ERK, Snail and E‐cadherin were detected by western blotting (*P < 0.05). (F) Serum‐starved SMMC‐7721 and HepG2 cells pre‐incubated with MK2206 were treated with HGF (10 ng·mL^−1^) and P‐AKT, Snail and E‐cadherin were detected by western blotting (*P < 0.05). (G) Serum‐starved SMMC‐7721 and HepG2 cells pre‐incubated with S3I‐201 were treated with HGF (10 ng·mL^−1^) and P‐STAT3, Snail and E‐cadherin were detected by western blotting (*P < 0.05). Data are expressed as the mean ± SD from three individual experiments. Differences between groups were determined using Student's *t*‐test and two‐way ANOVA with Bonferroni correction.

To further investigate the mechanism responsible for HGF‐induced sorafenib resistance, we focused on the three downstream pathways of HGF/MET signaling, namely the mitogen‐activated protein, phosphoinositide 3‐kinase (PI3K)/Akt and STAT3 pathways. We first detected P‐ERK, P‐Akt and P‐STAT3 activation in HCC cells with HGF stimulation. We found that HGF activated P‐ERK, P‐Akt and P‐STAT3 dose‐dependently (Fig. 4A) at 3 h after incubation. The time‐course study revealed that HGF activated P‐MET, P‐ERK, P‐Akt and P‐STAT3 as early as 1 h after stimulation (Fig. 4B). To determine whether P‐ERK, P‐Akt and P‐STAT3 activation induced sorafenib resistance, we used the U0126 (inhibitor of P‐ERK), MK2206 (inhibitor of P‐Akt) and S3I‐201 (inhibitor of P‐STAT3) in further investigations. Serum‐starved HCC cells were pre‐incubated with the inhibitors for 6 h before stimulation of HGF, and then the cells were administrated sorafenib at different concentrations. All three inhibitors block the protective effect of HGF on cell viability inhibition (Fig. 4C,D). The western blotting results showed that U0126 inhibited P‐ERK activation and Snail up‐regulation at 3 h. The down‐regulation of E‐cadherin was also reversed after inhibiting P‐ERK, indicating that P‐ERK inhibition reversed HGF‐induced EMT (Fig. 4E). Similar results were found in HCC cells treated with P‐STAT3 inhibitor S3I‐201. The administration of S3I‐201 reversed HGF‐induced EMT (Fig. 4G). The P‐Akt inhibitor MK2206 inhibited HGF‐induced sorafenib resistance but had no effect on HGF‐induced Snail up‐regulation and E‐cadherin down‐regulation (Fig. 4F). These results demonstrate that inhibition of P‐ERK, P‐Akt and P‐STAT3 inhibited HGF‐induced sorafenib resistance, and that P‐ERK and P‐STAT3 activation was responsible for HGF‐induced EMT.

### Regorafenib reverses HGF‐induced sorafenib resistance in HCC cells

**Figure 5:**
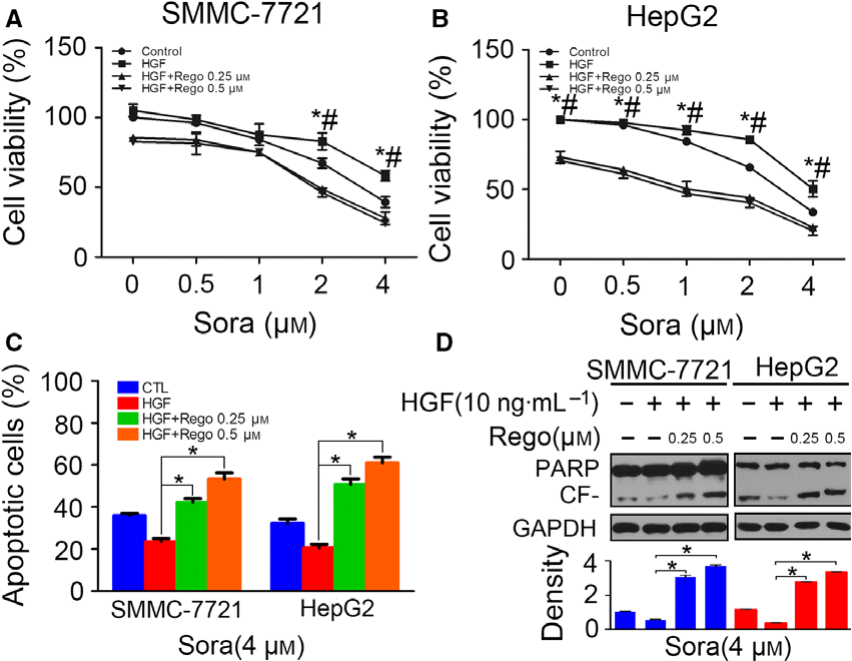
Regorafenib reverses HGF‐induced sorafenib resistance in HCC cells. (A and B) Serum‐starved SMMC‐7721 and HepG2 cells pre‐incubated with regorafenib (0.25 μm or 0.5 μm) for 6 h were treated with HGF (10 ng·mL^−1^) for 24 h and, thereafter, cells were incubated with sorafenib for 48 h. Cell viability was accessed by the CCK‐8 assay (*P < 0.05, HGF+Rego 0.25 μm vs. HGF; #*P* < 0.05, HGF + Rego 0.5 μm vs. HGF). (C) Following the treatment as described above, HCC cells treated with sorafenib (4 μm) were subjected to flow cytometry analysis, and apoptotic cells were compared (*P < 0.05). (D) Following the treatment as described above, PARP were detected by western blotting in HCC cells treated with sorafenib of 4 μm. The density of each band was normalized to GAPDH (*P < 0.05). Data are expressed as the mean ± SD from three individual experiments. Differences between groups were determined using Student's *t*‐test and two‐way ANOVA with Bonferroni correction.

Regorafenib is a multitargeted tyrosine kinase inhibitor and was recently approved as the second‐line therapy of advanced HCC patients who progressed on sorafenib treatment 13. We examined whether regorafenib could inhibit HGF‐induced sorafenib resistance. HCC cells were pre‐treated with regorafenib for 6 h before HGF stimulation and sorafenib incubation, and then cell viability was assessed. We found that regorafenib inhibited the increase of cell viability after HGF stimulation in SMMC‐7721 (Fig. 5A) and HepG2 cells (Fig. 5B). A flow cytometry assay also demonstrated that regorafenib increased apoptosis in HGF‐treated HCC cells (Fig. 5C). The western blotting results showed the reversal of cleaved PARP decrease (Fig. 5D). The above results indicated that pre‐treatment of regorafenib reversed HGF‐induced sorafenib resistance in HCC cells.

### Regorafenib inhibits ERK and STAT3 activation

**Figure 6:**
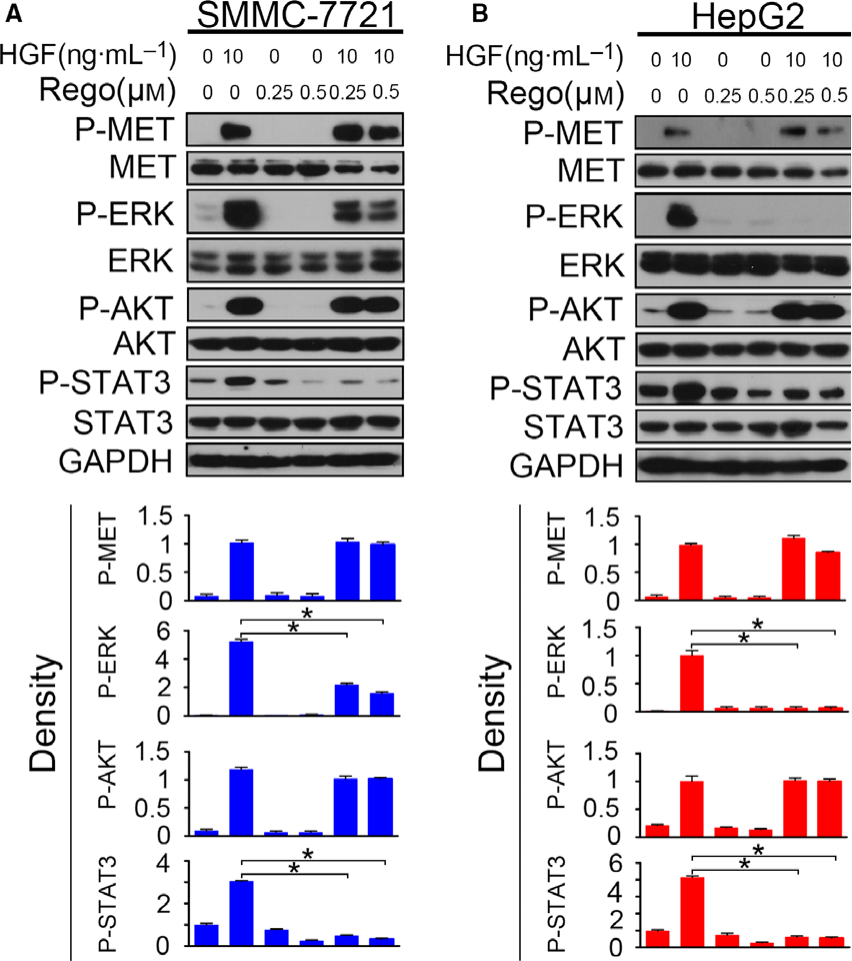
Regorafenib inhibits ERK and STAT3 activation. (A and B) Serum‐starved SMMC‐7721 and HepG2 cells pre‐incubated with regorafenib (0.25 μm or 0.5 μm) for 6 h were treated with HGF (10 ng·mL^−1^) for 1 h, and then P‐MET, P‐ERK, P‐AKT and P‐STAT3 were detected by western blotting. The density of each band was normalized to GAPDH (*P < 0.05). Data are expressed as the mean ± SD from three individual experiments. Differences between groups were determined using Student's *t*‐test and two‐way ANOVA with Bonferroni correction.

To investigate the mechanism by which regorafenib negatively regulated HGF‐induced sorafenib resistance, we examined the HGF/MET signaling after regorafenib and HGF treatment. HCC cells pre‐incubated with regorafenib were incubated with HGF for 3 h, and then P‐MET, P‐ERK, P‐Akt and P‐STAT3 were evaluated by western blotting. The results showed that regorafenib influenced the downstream signaling of HGF/MET without affecting P‐MET activation. Regorafenib had a competent inhibitory role on P‐ERK and P‐STAT3, although it had no effect on P‐Akt activation. Decreased activation of P‐ERK and P‐STAT3 was observed at HCC cells with regorafenib treatment; however, the protein levels of P‐Akt remained the same (Fig. 6).

### Regorafenib inhibits EMT by down‐regulating Snail in HCC cells

**Figure 7:**
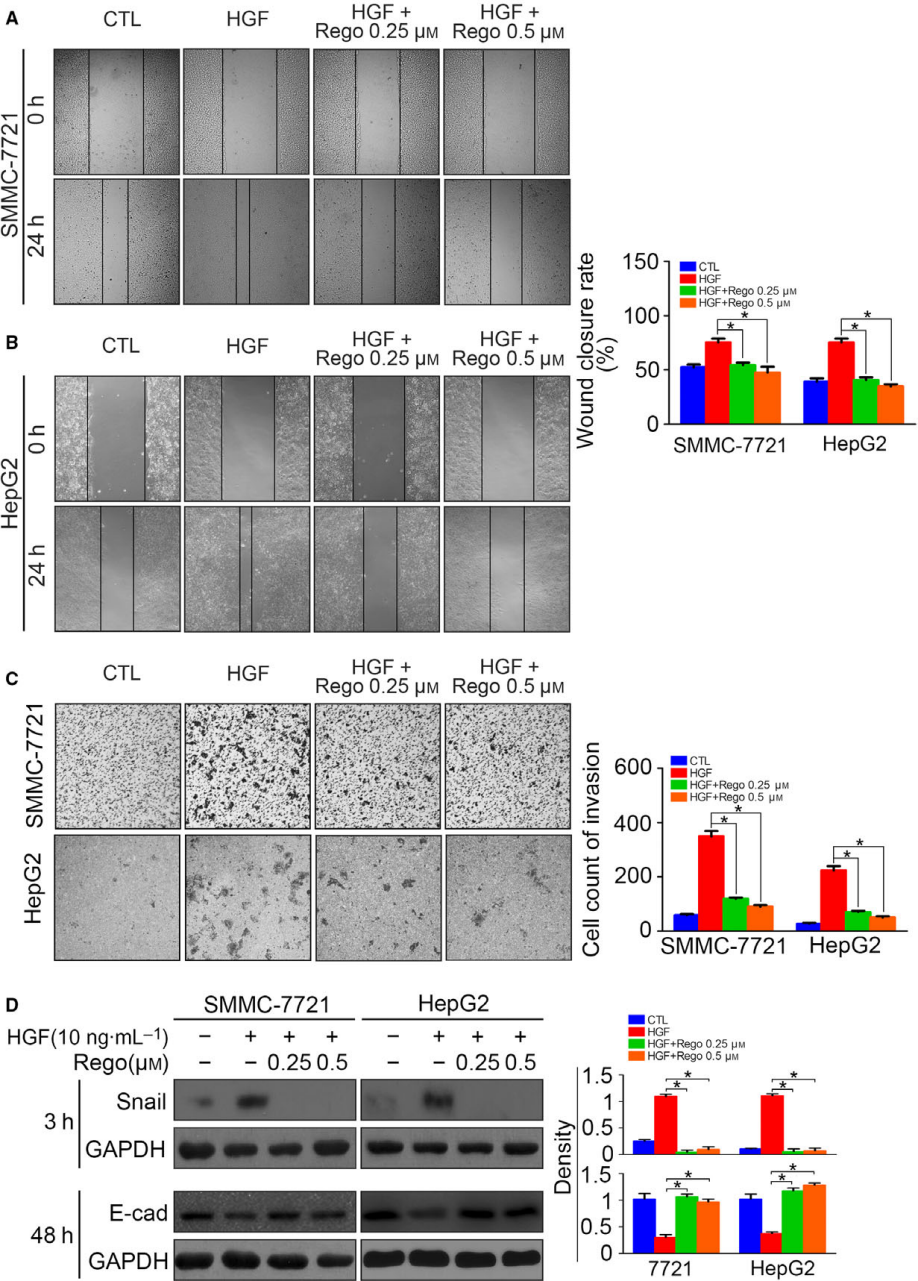
Regorafenib inhibits EMT by down‐regulating Snail in HCC cells. (A) Serum‐starved SMMC‐7721 cells pre‐incubated with regorafenib (0.25 μm or 0.5 μm) for 6 h were treated with HGF (10 ng·mL^−1^) for 24 h, and cell migration was determined by a wound healing assay. (B) Serum‐starved HepG2 cells pre‐incubated with regorafenib (0.25 μm or 0.5 μm) for 6 h were treated with HGF (10 ng·mL^−1^) for 24 h, and cell migration was determined by a wound healing assay. (C) Serum‐starved SMMC‐7721 and HepG2 cells pre‐incubated with regorafenib (0.25 μm or 0.5 μm) for 6 h were treated with HGF (10 ng·mL^−1^) for 24 h, and cell invasion was determined by a transwell assay. (D) Serum‐starved SMMC‐7721 and HepG2 cells pre‐incubated with regorafenib (0.25 μm or 0.5 μm) for 6 h were treated with HGF (10 ng·mL^−1^) for 1 h, and Snail was detected 3 h after treatment, whereas E‐cadherin was detected 48 h after treatment, by western blotting. The density of each band was normalized to GAPDH (*P < 0.05). Data are expressed as the mean ± SD from three individual experiments. Differences between groups were determined using Student's *t*‐test and two‐way ANOVA with Bonferroni correction.

As demonstrated above, Snail up‐regulation following P‐ERK and P‐STAT3 activation was the reason for HGF‐induced sorafenib resistance. Accordingly, we attempted to clarify whether regorafenib reversed HGF‐induced EMT. Serum‐starved HCC cells pre‐treated with regorafenib were stimulated with HGF for 24 h, and the migration capacity was accessed using a wound closure assay. We found that regorafenib inhibited the HGF‐induced cell migration (Fig. 7A,B). Using a transwell assay, we also observed impaired invasion following regorafenib treatment in HCC cells with HGF stimulation (Fig. 7C). The western blotting results showed that regorafenib inhibited HGF‐induced Snail up‐regulation and E‐cadherin down‐regulation. These results confirmed that regorafenib down‐regulated Snail, and thus inhibited EMT in HGF‐treated HCC cells.

## Discussion

Innate and acquired sorafenib resistance limits its efficiency and is a major obstacle towards achieving a better outcome in advanced HCC patients 6. Much concern has been raised worldwide regarding the underlying mechanisms. In the present study, we focused on HGF‐induced sorafenib resistance and attempted to reveal the possible mechanisms and also determine whether regorafenib could reverse sorafenib resistance.

Soluble factors such as cytokines and growth factors from paracrine or autocrine sources in the tumor microenvironment are a major cause of acquired resistance against targeted therapies 11. Among the various factors, HGF confers substantial resistance to rapidly accelerated fibrosarcoma (RAF) and mitogen‐activated protein kinase kinase (MEK) inhibition by activation of the HGF/MET axis 20. The activation of HGF/MET pathway was commonly found with respect to sorafenib resistance in HCC cells. A study by Firtina *et al*. 21 showed that sorafenib‐resistant HCC cells demonstrated induction of HGF synthesis and secretion, as well as increased levels of MET kinase activation, which indicated an autocrine activation of HGF/MET signaling. Han *et al*. 22 also reported HGF overexpression and enhanced MET activation in sorafenib‐resistant HCC cells. Thus, in the present study, we focused on sorafenib resistance induced by HGF.

Upon HGF stimulation, the downstream ERK and STAT3 were found to be activated in HCC cells. The inhibition of ERK and STAT3 activation reversed HGF‐induced sorafenib resistance. Because sorafenib is designed to block the RAF/MEK/ERK pathway and induce tumor inhibition in HCC, the reactivation of ERK pathway confers an acquired resistance to BRAF and MEK inhibitors 23. The STAT3 activation was also a mediator of sorafenib resistance. Su *et al*. 24 found that sustained sorafenib treatment inactivated regulatory factor SH2 domain‐containing phosphatase 1 (SHP‐1) and further up‐regulated P‐STAT3, which exerted sorafenib resistance. However, how activated ERK and STAT3 confers sorafenib resistance remains to be clarified. We found down‐regulated E‐cadherin and up‐regulated vimentin in HCC cells and the cell migration and invasion was enhanced following HGF treatment. HGF induced EMT in HCC cells by increasing Snail. The inhibition of ERK and STAT3 reversed Snail up‐regulation. In HepG2 cells, we observed continuous activation of P‐STAT3 after 6 h of HGF stimulation; however, Snail decreased after 6 h and P‐ERK activation also started to decline in the same trend. The continuous activation of P‐STAT3 might be the result of the activation of some certain feedback loops, such as the COX‐2/PGE2/STAT3 loop 25 and the interleukin‐6/STAT3 axis 26. The continuous activation of P‐STAT3 did not up‐regulate Snail persistently; thus, we speculated that the MET/ERK/Snail/EMT pathway was the major pathway in HGF‐induced EMT. EMT is a multistep cellular reprogramming process by which epithelial cells turn into mesenchymal types 27. Several studies have demonstrated EMT is implicated in sorafenib resistance in HCC 28, 29, 30. Zhang *et al*. 29 found that galectin‐1 overexpression induced HCC cell EMT via the PI3K/Akt pathway. Research by Huang *et al*. 28 demonstrated that αB‐crystallin overexpression induced EMT in HCC cells via activation of the ERK cascade. Bae *et al*. 30 found that HCC cells with higher serum response factor expression demonstrated mesenchymal phenotypes and were less responsive to sorafenib‐mediated apoptotic effect. The expression of serum response factor was significantly correlated with EMT transcription factor Snail 30.

We also found Akt activation following HGF stimulation. Inhibition of Akt by MK2206 reversed HGF‐induced sorafenib resistance but had no effect on Snail protein level. This finding was consistent with the results of a study by Nagai *et al*. 19 showing that HGF induced EMT via the ERK pathway but not the Akt pathway in HCC cells. The activated PI3K/Akt pathway might promote sorafenib resistance via other mechanisms instead of EMT. It was reported that the PI3K/Akt pathway had a vital role in survival and proliferation, and its alteration in cancer cells exploits normal mechanisms to overcome apoptosis 31. Our previous reports showed that the activated PI3K/Akt pathway mediated acquired resistance to sorafenib in HCC by increasing the anti‐apoptotic protein Mcl‐1 and Bcl2 levels 12. HGF‐induced sorafenib resistance could be attributed to the combined action of EMT and other anti‐apoptotic and pro‐proliferative mechanisms.

The inhibitory effect of regorafenib on sorafenib resistance has not yet been reported. In our research, we found that regorafenib reversed sorafenib resistance by inhibiting the ERK and STAT3 pathway and the subsequent Snail up‐regulation and EMT. The inhibition on ERK activation was consistent with previous studies showing that regorafenib was designed to inhibit the RAF/MEK/ERK pathway 17. The mechanism by which STAT3 activation was inhibited by regorafenib was first reported by Fan *et al*. 32 in colorectal cancer cells. They found that regorafenib significantly enhanced SHP‐1 activity, which dramatically decreased the phosphorylated form of STAT3 at Tyr705. Regorafenib also augmented SHP‐1 activity by direct disruption of the association between N‐SH2 and catalytic PTP domain of SHP‐1. The inhibition of STAT3 by regorafenib induced colorectal cancer cell growth inhibition 32. A similar mechanism was also discovered in their researches into EMT of colorectal cancer cells. They found that regorafenib exerted potent inhibitory effect on TGF‐β1‐induced EMT by enhancing SHP‐1 activity and inhibiting P‐STAT3 33. In HCC cells, regorafenib also induced tumor growth inhibition by relieving the autoinhibited N‐SH2 domain of SHP‐1 directly and inhibiting P‐STAT3 signals 15. The mechanism by which P‐STAT3 was inhibited in EMT of HCC cells was not investigated in the present study. The down‐regulated P‐STAT3 might also be a consequence of enhanced SHP‐1 activity.

## Conclusions

In conclusion, our research in the present study has demonstrated that HGF induced sorafenib resistance by activating the P‐ERK/Snail/EMT and P‐STAT3/Snail/EMT pathway. The inhibition of P‐ERK and P‐STAT3 by regorafenib could block HGF‐induced EMT and thus reverse HGF‐induced sorafenib resistance. PI3K/Akt signaling also participated in HGF‐induced sorafenib resistance, probably by promoting survival and proliferative pathways. The combination of regorafenib and P‐Akt inhibitors might have a potent inhibitory effect on sorafenib‐resistant HCC cells.

## Conflicts of interest

The authors declare no conflict of interest.

## Author contributions

WC and JY conducted the experiments and wrote the paper. YZ and HC analyzed and interpreted the data. XC and DS conceived and designed the project.
