# Corrigendum to: Regorafenib reverses HGF‐induced sorafenib resistance by inhibiting epithelial‐mesenchymal transition in hepatocellular carcinoma

**DOI:** 10.1002/2211-5463.13403

**Published:** 2022-04-18

Weibo Chen, Junsheng Yang, Yue Zhang, Huihua Cai, Xuemin Chen, Donglin Sun

The original article [1] contained unnoticed mistakes in the western blots, transwell, and wound‐healing assays presented in Figures 2 and 7. Corrected versions of Figures 2 and 7 are presented below. The transwell assay presented in Figures 2B and 7C are the same experiment, and the control presented for the HepG2 cells is the same between these two figures. The conclusions were unaffected by the errors in the preparation of the figures and are still supported by the corrected data.

**Fig. 2:**
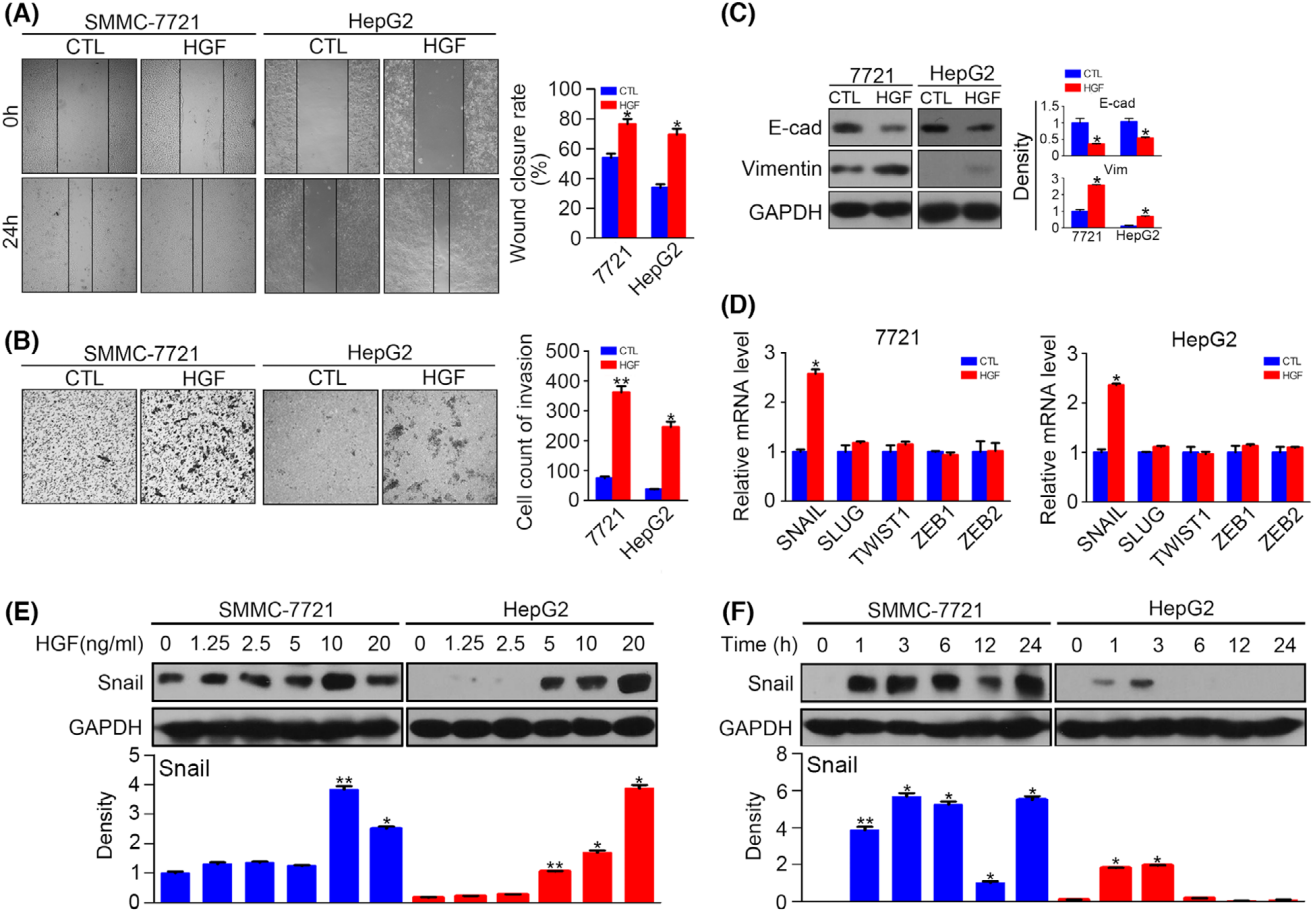
Hepatocyte growth factor induces EMT by upregulating Snail in HCC cells. (A) Serum‐starved SMMC‐7721 and HepG2 were stimulated with or without HGF (10 ng·mL^−1^) for 24 h, and then, cell migration was determined by a wound‐healing assay. (B) Serum‐starved SMMC‐7721 and HepG2 were stimulated with or without HGF (10 ng·mL^−1^) for 24 h, and then, cell invasion was determined by a transwell assay. This is the same experiment as that presented in Figure 7C, and the control presented for the HepG2 cells is the same between these two figures. (C) Serum‐starved SMMC‐7721 and HepG2 were stimulated with or without HGF (10 ng·mL^−1^) for 48 h, and the protein levels of E‐cadherin and vimentin were detected by western blotting. The density of each band was normalized to GAPDH. (D) Quantitative RT‐PCR results of snail, slug, twist1, zeb1, and zeb2 after incubation with HGF for 3 h. (E) Serum‐starved SMMC‐7721 and HepG2 were stimulated with HGF at different concentrations for 3 h, and protein levels of Snail were detected by western blotting. The density of each band was normalized to GAPDH. (F) Serum‐starved SMMC‐7721 and HepG2 were stimulated with HGF (10 ng·mL^−1^) for different times, and protein levels of Snail were detected by western blotting. The density of each band was normalized to GAPDH (*P < 0.05, **P < 0.01, compared to control). Data are expressed as the mean ± SD from three individual experiments. Differences between groups were determined using the Student's *t*‐test and two‐way ANOVA with Bonferroni correction.

**Fig. 7:**
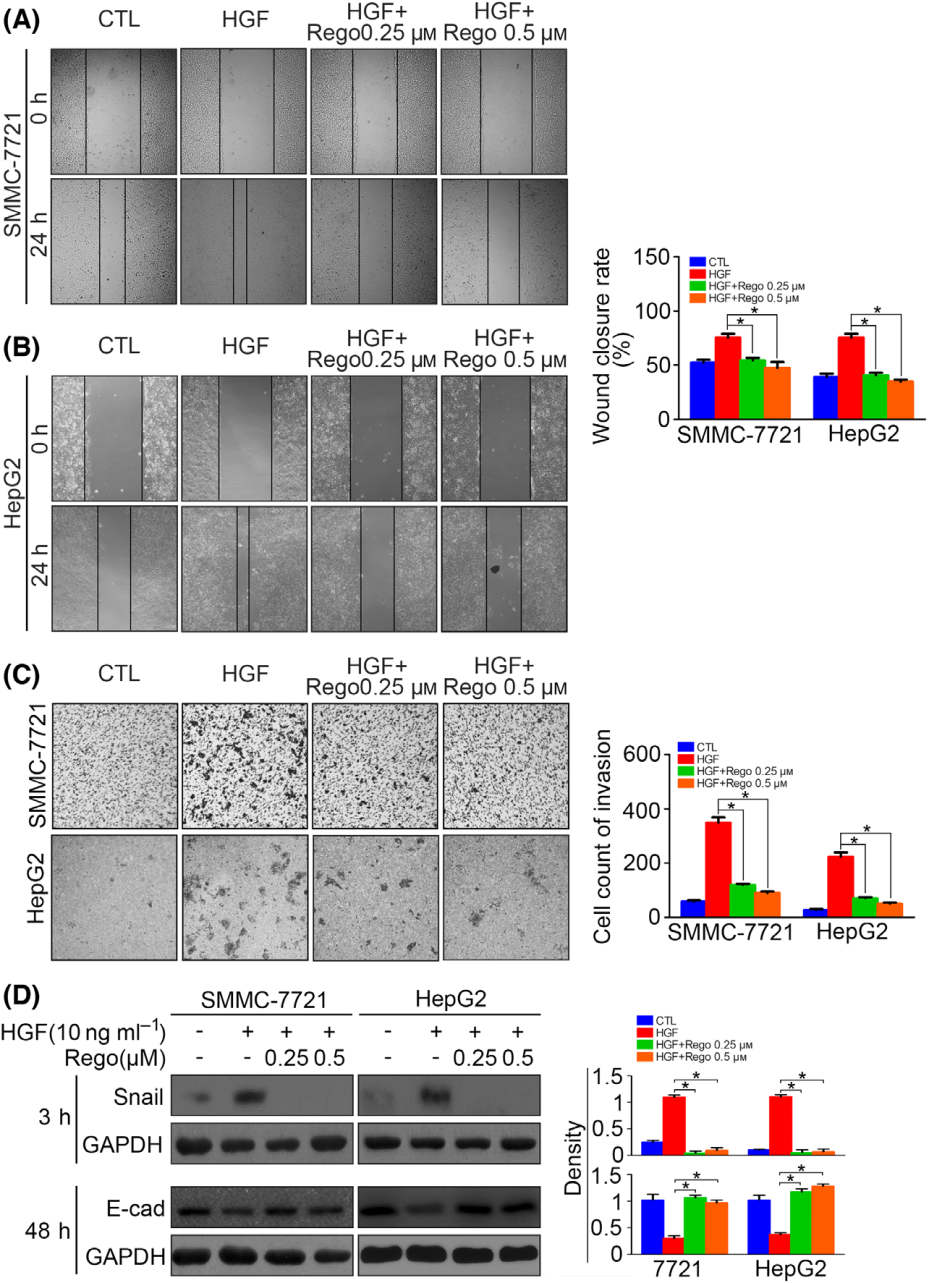
Regorafenib inhibits EMT by downregulating Snail in HCC cells. (A) Serum‐starved SMMC‐7721 cells preincubated with regorafenib (0.25 μm or 0.5 μm) for 6 h were treated with HGF (10 ng·mL^−1^) for 24 h, and cell migration was determined by a wound‐healing assay. (B) Serum‐starved HepG2 cells preincubated with regorafenib (0.25 μm or 0.5 μm) for 6 h were treated with HGF (10 ng·mL^−1^) for 24 h, and cell migration was determined by a wound‐healing assay. (C) Serum‐starved SMMC‐7721 and HepG2 cells preincubated with regorafenib (0.25 μm or 0.5 μm) for 6 h were treated with HGF (10 ng·mL^−1^) for 24 h, and cell invasion was determined by a transwell assay. This is the same experiment as that presented in Figure 2B, and the control presented for the HepG2 cells is the same between these two figures. (D) Serum‐starved SMMC‐7721 and HepG2 cells preincubated with regorafenib (0.25 μm or 0.5 μm) for 6 h were treated with HGF (10 ng·mL^−1^) for 1 h, and Snail was detected 3 h after treatment, whereas E‐cadherin was detected 48 h after treatment, by western blotting. The density of each band was normalized to GAPDH (*P < 0.05). Data are expressed as the mean ± SD from three individual experiments. Differences between groups were determined using the Student's *t*‐test and two‐way ANOVA with Bonferroni correction.
